# Patterns of cross-contamination in a multispecies population genomic project: detection, quantification, impact, and solutions

**DOI:** 10.1186/s12915-017-0366-6

**Published:** 2017-03-29

**Authors:** Marion Ballenghien, Nicolas Faivre, Nicolas Galtier

**Affiliations:** 1 UMR5554 – Institute of Evolutionary Sciences, University Montpellier, CNRS, IRD, EPHE, Place Eugène Bataillon, CC64, 34095 Montpellier, France; 2 UMR7144 - Adaptation et Diversité en Milieu Marin - CNRS, Université Pierre et MarieCurie, Station Biologique de Roscoff, 29680 Roscoff, France

**Keywords:** RNAseq, Transcriptome, Animals, SNP calling, Genotyping, Within-species

## Abstract

**Background:**

Contamination is a well-known but often neglected problem in molecular biology. Here, we investigated the prevalence of cross-contamination among 446 samples from 116 distinct species of animals, which were processed in the same laboratory and subjected to subcontracted transcriptome sequencing.

**Results:**

Using *cytochrome oxidase* 1 as a barcode, we identified a minimum of 782 events of between-species contamination, with approximately 80% of our samples being affected. An analysis of laboratory metadata revealed a strong effect of the sequencing center: nearly all the detected events of between-species contamination involved species that were sent the same day to the same company. We introduce new methods to address the amount of within-species, between-individual contamination, and to correct for this problem when calling genotypes from base read counts.

**Conclusions:**

We report evidence for pervasive within-species contamination in this data set, and show that classical population genomic statistics, such as synonymous diversity, the ratio of non-synonymous to synonymous diversity, inbreeding coefficient F_IT_, and Tajima’s D, are sensitive to this problem to various extents. Control analyses suggest that our published results are probably robust to the problem of contamination. Recommendations on how to prevent or avoid contamination in large-scale population genomics/molecular ecology are provided based on this analysis.

**Electronic supplementary material:**

The online version of this article (doi:10.1186/s12915-017-0366-6) contains supplementary material, which is available to authorized users.

## Background

Contamination is a well-known and ancient problem in molecular biology research. Most people who have worked in a molecular biology laboratory for a while have at least once observed an extra band on a gel, or obtained DNA sequence data originating from an unexpected species. Projects involving a polymerase chain reaction (PCR) step are particularly sensitive to contamination because initially small amounts of foreign DNA can accidentally be amplified by PCR, and transferred from tube to tube. A number of published results obtained by Sanger sequencing were subsequently demonstrated to most likely result from laboratory contamination [1–4].

Massive sequencing projects and databases based on next-generation sequencing (NGS) technologies are far from immune from contamination issues [5–8]. The problem is perhaps even exacerbated, with contaminant sequence reads being lost in the myriad of reads from the target sample, and therefore difficult to detect and clean out. The concern is particularly serious when the target sample is from a non-model species lacking a reference genome, so that genuine sequence reads cannot be easily identified by similarity. It should be noted that many NGS library construction protocols involve one or multiple PCR amplification steps that generate elevated concentrations of DNA, thereby increasing the risk of contamination.

Contamination is a well-identified problem in projects targeting very small amounts of DNA, such as ancient DNA projects [9, 10] and low-frequency variant analysis [11–13], in which small amounts of contamination can be sufficient to confound the results. A couple of recent studies, however, have revealed that both cross-contamination [14] and environmental contamination [15] can cause serious problems even in standard NGS projects, that is, when target DNA is a priori thought to be much more abundant than contaminant DNA [16]. These studies ring an alarm bell and call for a systematic examination of the prevalence of contamination in past, present and future NGS datasets.

Recently, we conducted a multispecies population genomic project in which one to ten individuals from each of >100 non-model species of animals were subjected to RNA sequencing (RNAseq), leading to a number of scientific publications [17–30]. The co-occurrence in the same laboratory of many samples from many distinct species in a relatively short period of time provides an ideal situation for investigating the effect of cross-contamination in a molecular ecology project. Our goals in this study were multiple. First, we aimed to quantify the prevalence of cross-contamination, identifying at which steps of the experimental protocol it most often happens, and if possible delivering guidelines on how to avoid it. Second, we wanted to check the robustness of our published results to the problem of contamination, and if possible identify solutions to this problem.

Two distinct, complementary approaches were taken. Regarding between-species contamination, cytochrome oxidase 1 (*cox1*) was used to detect the occurrence of foreign cDNA sequences in a sample and trace their likely sources. *cox1* is a high-expressed gene, and is therefore expectedly prevalent in RNAseq data. It is the standard DNA barcoding tool in animals, so a huge database of *cox1* reference sequences from many distinct species of animals is available. Regarding within-species contamination, patterns of read counts were analyzed to search for evidence of allele leakage across individuals. The inferred patterns of contamination across individuals and species were considered in the light of laboratory metadata – dates of entry and processing of samples in the laboratory, identity of technicians in charge of the samples, date of shipment to sequencing center, identity of sequencing center, flowcell number, and lane number. A modified single nucleotide polymorphism (SNP)-calling method that accounts for among-individual contamination was introduced and a re-analysis of our main published results was conducted.

## Methods

### Project overview and protocols

European Research Council project 232971 “PopPhyl” took place at the Institute of Evolutionary Sciences Montpellier, France, from June 2009 to December 2014. During this period, samples from >3800 distinct individuals of 180 species from eight phyla of animals entered the laboratory located in building 32 of University Montpellier, France. Samples were either collected by ourselves in the field or shipped by colleagues in RNAlater® (Qiagen, Dusseldorf, Germany) buffer. A fraction of the samples were barcoded after DNA extraction and *cox1* amplification. Roughly 1200 samples were subjected to RNA isolation following standard or modified protocols [31]. The quality and quantity of extracted RNA were assessed using spectrophotometry and capillary electrophoresis. Total RNA from 446 of these samples was sent out for Illumina sequencing on either a Genome Analyzer II (2009–2010) or a HiSeq 2000 (2011–2014). Illumina library construction, DNA fragment tagging, pooling, and demultiplexing were achieved in the sequencing centers. Short-read data were returned to us as one or several FASTQ files per sample and we performed the downstream bioinformatic analyses [18]. No more than one sample per individual was sent out for sequencing. The individual samples that were sent out for sequencing belonged to 116 distinct species. Sixty-three additional species were subjected to RNA extraction but not sent out for Illumina sequencing.

In our laboratory, eight distinct persons, referred to below as “technicians,” processed the samples. Two technicians collectively processed ~80% of the samples. These two technicians, and the majority of the other technicians involved, were 100% dedicated to the project and did not (or very rarely) manipulate biological material coming from species not included in the project. In 141 species, the same technician processed all the samples, whereas in 39 species, two distinct technicians were involved. All samples were processed at the same laboratory bench, in a room almost entirely dedicated to the project, with specific materials shared by the involved technicians.

Samples were sent to sequencing centers in dry ice at 15 distinct dates, from September 23, 2009, to February 13, 2014. Shipments typically involved several individuals from several distinct species. In each shipment, samples were contained in separate, labeled tubes that were gathered in a single box and accompanied by a form briefly describing the label and content of each tube. Tubes in boxes were organized by species and ordered consistently with the form. When more than one technician was involved in a shipment, tubes were ordered by technician, that is, samples processed by technician 1 first, then samples processed by technician 2. Tubes were not opened at shipment stage: they were simply taken out from freezers, packed, and transferred to the carrier. Samples were sent to three distinct sequencing centers, of which one (SC1) processed ~85% of the samples. The dates of first entry in the laboratory, first and last experiment in the laboratory, and shipment(s) were recorded per species. This information is available in Additional file 1: Table S1. Details on experimental dates per sample are available on request. We also retrieved flowcell identifiers and lane numbers from read headers in FASTQ files – this information was absent from 88 of the files we received, though. We have no information on the dates of library construction and the identity of the technicians involved in library construction and sequencing.

### Short-read data sets

We analyzed Illumina short-read data sets from 446 individuals of 116 species (1–11 individuals per species; Additional file 1: Table S1). Read length was 100 in 358 individuals (92 species), 75 in 12 individuals (three species), and 50 in 76 individuals (21 species). Single-end sequencing was ordered in all cases. Occasionally, sequencing centers still returned paired-end reads, which were treated as single-end reads in our analyses for the sake of homogeneity – meaning that both reads were treated as independent events. The total number of reads varied from 2.44 to 76 millions among samples. Samples sequenced later in the project typically received more reads than early-sequenced samples. Most of the generated data sets have been submitted to the National Center for Biotechnology Information (NCBI) Sequence Reads Archive (SRA) under bioprojects PRJNA230239, PRJNA249058, PRJNA268920, PRJNA278516, PRJNA322119, and PRJNA326910. We here publish data from an additional 27 individuals from 19 species, which were submitted to NCBI-SRA under bioproject PRJNA374528. A list of sequenced individuals with associated identifiers is provided in Additional file 2: Table S2.

### *cox1* reference database

We created a reference database of *cox1* sequences for subsequent sequence similarity searches. This database had three components: a target species component, a companion species component, and a model species component. The target species component corresponds to *cox1* sequences from the species that have been subjected to DNA barcoding and/or RNA extraction in our laboratory between 2009 and 2014. We automatically downloaded target species *cox1* sequences from the Barcode Of Life Database [32] (May 6, 2016), accounting for taxonomic synonymy – we equated *Myodes* with *Clethrionomys*, *Cervus* with *Rucervus*, *Parus* with *Cyanistes*, *Mellicta* with *Melitaea*, *Physa* with *Physella*, *Galba* with *Lymnaea*, *Lineus* with *Ramphogordius*, and *Abatus agassizi* with *Abatus agassizii*. Sequences in the Barcode of Life Data System (bold) database are binned based on similarity. We kept a single *cox1* sequence per bin per target species, maximizing sequence length and number of annotations – geographic origin, collector, sampling date, lifestyle, tissue, and existence of voucher. Sequences not assigned to a bin were excluded. A similarity search was performed by BLAST to the NCBI non-redundant (NR) database. Thirteen sequences not hitting any *cox1* sequences from NR were removed or manually replaced. Some of our target species were not represented in the bold database. For these we performed a manual search in GenBank and retrieved additional *cox1* sequences.

The companion species component of our reference *cox1* database corresponds to species that never entered our laboratory, but that are phylogenetically related to target species. For each genus of our target species sample, we identified a companion genus from the same family or same order in which *cox1* sequences were available in a roughly equivalent number of species (Additional file 1: Table S1). The same automatic and cleaning procedure as described above for target species was applied to companion species, with the exception that we did not manually search GenBank for companion species. The companion species component was added as a negative control, that is, a measure of the prevalence of seemingly foreign *cox1* sequences in our samples due to experimental noise, in the absence of contamination.

The model species component of our reference *cox1* database corresponds to species of animals that are frequently subjected to NGS projects, with which our samples might have been in contact at some point during the experimental protocol – including *Homo sapiens*. We selected the 20 species of animals with the largest number of entries in the NCBI-SRA database (March 15, 2016), and retrieved the complete *cox1* sequence of each of these.

*cox1* sequences from the target species, companion species, and model species were aligned using MACSe [33]. A single segment of the *cox1* sequence was selected, from position 6189 to position 6539 (revised Cambridge reference sequence). Sequences for which the segment was not entirely determined were discarded. For each genus of the target species component, a phylogenetic tree was reconstructed using PHYML in SEAVIEW [34] and inspected by eye. Obvious anomalies were corrected by removing the misplaced sequences, in light of existing taxon-specific literature when available. Our reference *cox1* sequence alignment is provided as Additional file 3.

### Detection of between-species contamination

Short-read Illumina data sets were cleaned for low-quality reads or read portions as described previously [35]. Reads were mapped to the reference *cox1* database using Burrows-Wheeler Aligner (BWA) software [36]. Mapping tolerance was one mismatch for data sets in which read length was 50 and two mismatches for data sets in which read length was 75 or 100. The best scoring hit of each read, if any, was recorded – note that in case of equal mapping scores, BWA will randomly output one of the highest scoring hits. For each individual, the number of hits for each sequence of the reference database was recorded and normalized by total number of reads for the considered individual. The results were then summed up by species in order to calculate, for every pair of species (*sp1, sp2*), the total number of *cox1* reads from *sp1* mapping to a reference sequence from *sp2*. Species from the reference database between which *cox1* divergence was <5% were considered as non-diagnostic, meaning that a hit of a *sp1* read to a *sp2* read was only considered to reflect contamination if *cox1* divergence between *sp1* and *sp2* was >5%. When more than one reference sequence per species was available, we required that the minimal divergence between any two *cox1* sequences from the two species be >5%. Sixty such closely related species pairs were identified. These analyses were performed using homemade programs in C++ and R.

### Read counts, homo-quartets, and detection of within-species contamination

For each species including at least four individuals, transcriptome assembly was performed using a combination of ABySS [37] and CAP3 [38], as described previously [35]. Reads were mapped to predicted cDNAs using BWA, following [18] and [21], and potential PCR duplicates were removed – meaning that identical reads in any particular individual were counted only once. Open reading frames were predicted as in [18] and coding sequences were retained. For each position of each coding sequence and each individual, the number of reads for the four possible states A, C, G, and T were recorded. Below we refer to these vectors of counts as “quartets.”

**Fig. 1:**
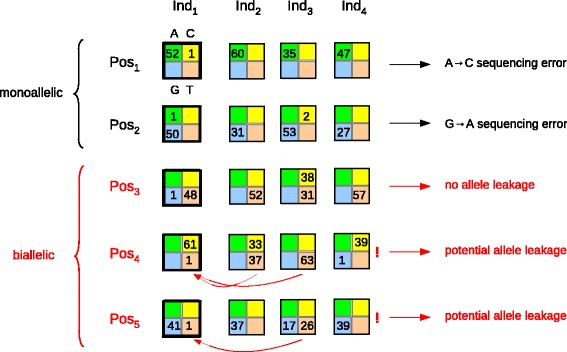
Detection of within-species contamination through homo-quartet analysis. Each multicolored square represents a quartet, that is, read counts for states A (*green*), C (*yellow*), G (*blue*), and T (*orange*) at a specific position in a specific individual, zeros being omitted. A fictive dataset of four individuals (*Ind*_*1*_ to *Ind*_*4*_) and five positions (*Pos*_*1*_ to *Pos*_*5*_) is shown. At all five positions, the quartet for individual Ind_1_ is a homo-quartet (*thick borders*): the major state has more than 40 reads, and the minor state has exactly one read. Positions Pos_1_ and Pos_2_ are monoallelic: the major state represents more than 95% of reads across the four individuals. These two positions inform on the contamination-free error pattern. Positions Pos_3_, Pos_4_, and Pos_5_ are biallelic: besides the major state, another allele segregates in the sample. At Pos_3_ the Ind_1_, the minor state (G) differs from the other segregating allele (C); this error cannot result from within-species contamination. At Pos_4_ and Pos_5_, the Ind_1_ minor state is identical to the other segregating allele (T), potentially reflecting allele leakage between individuals, as indicated by *red arrows*. The proportions of these different types of position inform on the prevalence of within-species contamination

To characterize within-species, between-individual contamination, we focused on quartets in which (1) exactly two states were observed, (2) read count was high (above 40) for the most prevalent state, and (3) read count was exactly one for the other state. Such quartets were assumed to correspond to genotypes that are homozygous for the major state and in which an error had been introduced – the minor state. We called these quartets “homo-quartets.” We defined two categories of homo-quartets, depending on the read counts in other individuals at the same position. The first category corresponds to homo-quartets occurring at positions dominated by the major state – specifically, when the sum across individuals of allele counts for the major state was >95% of total counts. Such positions were called monoallelic (Fig. 1, black). The second category corresponds to homo-quartets occurring at positions in which two alleles were found at a substantial frequency – specifically, when the sum across individuals of read counts for the second more frequent state was more than 10*n*, *n* being the number of genotyped individuals. Such positions were called biallelic (Fig. 1, red). Homo-quartets not falling in either of these two categories were disregarded.

We reasoned that, in the absence of contamination, the identity of the minor state in any given homo-quartet should be independent of read counts for other individuals, so that a similar pattern of sequencing error would be expected for the two categories of homo-quartets. If, however, the distribution of the minor state in homo-quartets differed between monoallelic and biallelic positions, and was influenced at biallelic positions by the identity of alleles segregating in the sample, then such a pattern would demonstrate the existence of within-species contamination (Fig. 1).

Formally, for each species, we first considered homo-quartets occurring at monoallelic positions and calculated **P**, the matrix of minor state prevalence given the major state:

1 $$ P\left( a, b\right)={h}_{mono}\left( a, b\right)/{\displaystyle \sum }{h}_{mono}\left( a, k\right) $$

where *a* and *b* are two of the four A, C, G, and T states (*b* ≠ *a*), and *h*_mono_(*a,b*) is the number of homo-quartets occurring at monoallelic positions and having *a* as the major state and *b* as the minor state. **P** can be understood as an estimate of the error matrix at monoallellic positions.

Then we considered homo-quartets occurring at biallelic positions and calculated *q*_*obs*_, the observed prevalence as a minor state of the other allele segregating at the considered position:

2 $$ {q}_{obs}={\displaystyle \sum {r}_z}(k)/{h}_{bi} $$

where *h*_bi_ is the number of homo-quartets occurring at biallelic positions, *z* is the allele different from the major state segregating at the position at which homo-quartet *k* occurs, and *r*_*z*_(*k*) is read count for state *z* at homo-quartet *k*. By definition of homo-quartets, *r*_*z*_(*k*) must be zero or one. This number was compared to *q*_*exp*_, the expected prevalence as a minor state in homo-quartets of the other segregating allele assuming no contamination, that is, assuming that sequencing errors at homo-quartets occurring at biallelic positions are well predicted by **P**:

3 $$ {q}_{\exp }={\displaystyle \sum P}\left( a, z\right)/{\displaystyle \sum {\displaystyle \sum P}}\left( a, i\right) $$

where *a* is the major state of homo-quartet *k* and *z* is the allele different from *a* segregating at the position at which *k* occurs. We defined λ, the index of allele leakage among individuals within a species as:

4 $$ \lambda =\left({q}_{obs}{\textstyle \hbox{-} }{q}_{exp}\right)/{q}_{exp} $$

λ is expected to equal zero in the absence of contamination, that is, when the identity of the minor state for a given homo-quartet is independent of the genotypes and read counts of other individuals.

### Contamination-aware genotype calling

We modified our genotype-calling procedure [17, 18] to account for between-individual, within-species contamination. Following [17], we describe a quartet *R* by *r*_1_ (number of A reads at a given position for a given diploid individual), *r*_2_ (C reads), *r*_*3*_ (G reads), and *r*_4_ (T reads), and define *r* = *r*_1_ + *r*_2_ + *r*_3_+ *r*_4_. Let us call *f*_1_, *f*_2_, *f*_3_, and *f*_4_ the frequencies of alleles A, C, G, and T in the population at the considered position. Assuming Hardy-Weinberg equilibrium and a constant error rate ε, the probability of a quartet can be written as:

5 $$ \Pr (R)={\displaystyle \sum_{a=1}^4{\displaystyle \sum_{b= a}^4{f}_a{f}_b\frac{r!}{r_1!{r}_2!{r}_3!{r}_4!}{\displaystyle \prod_{x=1}^4{q}_x{\left[ ab\right]}^{r_x}}}} $$

with

6 $$ {q}_a\left[ aa\right]=1-3\upvarepsilon $$

7 $$ {q}_b\left[ aa\right]=\varepsilon $$

8 $$ {q}_a\left[ ab\right]=1/2-\varepsilon $$

9 $$ {q}_c\left[ ab\right]=\varepsilon $$

where *a ≠ b ≠ c* are in {A,C,G,T}. *q*_*x*_[*yz*] is the probability of calling state *x* from an individual carrying genotype {*y,z*}*.* Equations 5, 6, 7, 8, and 9 assume that, for every read, the genuine state will be called with probability 1 − 3ε, whereas an erroneous state will be called with probability ε. Equation 5 sums the contributions of the 10 possible diploid genotypes to the likelihood of *R*. In our implementation, the allele frequencies *f*_k_ were estimated from observed read counts, the error rate was estimated by maximizing the likelihood function, and distinct rates were assumed for transition-type vs. transversion-type errors [17].

Here, we introduce a generalization of Eqs. 6, 7, 8, and 9:

10 $$ {q}_a\left[ aa\right]=\left(1-\gamma \right)\left(1-3\upvarepsilon \right)+\gamma \left({f}_a\hbox{'}\left(1-3\upvarepsilon \right)+\left(1-{f}_a\hbox{'}\right)\varepsilon \right) $$

11 $$ {q}_b\left[ aa\right]=\left(1-\gamma \right)\varepsilon +\gamma \left({f}_b\hbox{'}\left(1-3\upvarepsilon \right)+\left(1-{f}_b\hbox{'}\right)\varepsilon \right) $$

12 $$ {q}_a\left[ ab\right]=\left(1-\gamma \right)\left(1/2-\varepsilon \right)+\gamma \left({f}_a\hbox{'}\left(1-3\upvarepsilon \right)+\left(1-{f}_a\hbox{'}\right)\varepsilon \right) $$

13 $$ {q}_c\left[ ab\right]=\left(1-\gamma \right)\varepsilon +\gamma \left({f}_c\hbox{'}\left(1-3\upvarepsilon \right)+\left(1-{f}_c\hbox{'}\right)\varepsilon \right) $$

where *f*_x_' is the frequency of reads of state *x* at the considered position excluding the focal individual. Equations 10, 11, 12, and 13 assume that with probability (1 − γ) a read state is determined by genotype and error rate, as in the original method, whereas with probability γ a read state is obtained by randomly sampling one state at the considered position, excluding reads from the focal individual. Here, γ is the probability of contamination, and is assumed to be homogeneously distributed across individuals of the sample. Note that even a contaminant read can be affected by sequencing error, as expressed in the right-hand term of Eqs. 10, 11, 12, and 13. Genotypes and SNPs were called assuming four distinct values for γ, namely γ = 0 (no contamination), γ = 0.05, γ = 0.1, and γ = 0.2.

## Results

### *cox1* reference database

We created a reference database of 624 aligned, partial, 351-bp-long *cox1* sequences. The database included a mixture of sequences from our target species (378 sequences from 149 species), companion species (226 sequences from 139 species), and model species (20 sequences from 20 species). Target species were intended to trace cross-contamination among samples. Companion species were introduced as negative controls. Model species were introduced to search for contamination by standard laboratory organisms. In our reference databases, 31 of our target species were not represented at all, 98 were represented by a single *cox1* sequence, and six were represented by more than ten *cox1* sequences, implying that our ability to detect the occurrence of a given species in a given sample varied among species.

### Patterns of between-species contamination

Short sequence reads from each of 446 samples (individuals) from 116 species were aligned to our reference *cox1* database using BWA. The number of hits to each reference sequence was recorded and divided by the number of millions of reads of the considered sample. For each sample, we calculated the prevalence of *cox1* hits to a reference sequence from the expected species, and the prevalence of *cox1* hits to a reference sequence from an unexpected species – that is, a species differing from the expected species by >5% of *cox1* divergence. Hits to a species different but <5% divergent from the expected one were not counted.

**Fig. 2:**
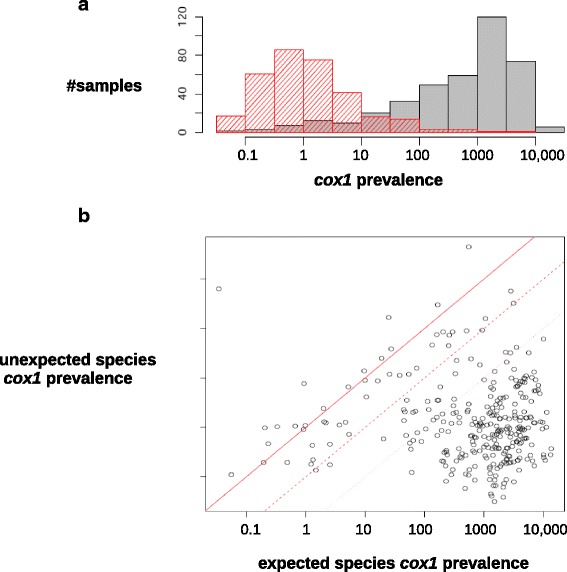
Overall pattern of between-species contamination. **a** Among-sample distribution of the prevalence of reads mapping to a *cox1* reference from the expected (*gray*) or an unexpected (*red*) species. Prevalence is defined as the number of *cox1* reads per million reads. **b** Relationship between the prevalence of *cox1* reads mapping to the expected (*x*-axis) vs. an unexpected (*y*-axis) species, again per million reads. Each *dot* represents a sample. *Plain line*: ratio of unexpected to expected *cox1* reads is one. *Dotted lines*: ratio of unexpected to expected *cox1* reads is 0.1 (respectively, 0.01). Samples from species not represented in our *cox1* reference database are not shown

Figure 2 shows an overview of the contamination pattern in this large-scale data set. Figure 2a shows the across-samples distribution of the prevalence of expected (gray) vs. unexpected (red) *cox1* reads, while Fig. 2b plots these two variables. The across-samples median prevalence of expected *cox1* reads was 674 *cox1* reads per million. The prevalence of expected *cox1* reads was sometimes low: it was <10 per million in 86 samples, and zero in 52 samples, of which 13 were from a species that was represented in our reference *cox1* database. This is quite surprising, given that *cox1* is considered a generally high-expressed gene. This result might be explained by insufficient/inappropriate species representation in the reference database for these particular samples. It might also be that in some taxa mitochondrial transcripts lack a polyA tail (or use it as a degradation signal, as in plants [39]) and were therefore excluded at the retrotranscription stage in our protocol.

We found at least one hit to an unexpected species in 353 of the 446 samples. The prevalence of unexpected *cox1* hits was >50 per million in 22 samples, and >500 per million in seven samples. One species, woodlouse *Armadillidium vulgare*, was particularly affected by unexpected hits – six individuals out of ten showed >50 per million unexpected hits. Twelve samples for which the prevalence of expected hits was >100 per million had a ratio of unexpected to expected hits >0.1, and two samples, GA24O (earthworm *Allolobophora chlorotica* L1) and GA17L (brine shrimp *Artemia tibetiana*), had a ratio >1.0. In summary, expected *cox1* reads clearly dominated but contaminant reads were common and reached a high prevalence in a substantial number of samples.

The vast majority (99.54%) of the 385,597 unexpected *cox1* reads originated from target species. Only 0.11% of the unexpected hits were assigned to a companion species, and 0.35% to a model species. The low prevalence of companion species was expected and confirmed that unexpected *cox1* hits result almost uniquely from contamination. Regarding model species, we detected human *cox1* reads in ten samples from nine distinct species, but always at very low prevalence – the total number of reads hitting a human *cox1* sequence was 92. *Mus musculus* and *Bos taurus* were more prevalent in terms of total reads (507 and 447, respectively), but concerned a smaller number of samples (five and three) and species (three and three, respectively).

Among the 446 analyzed samples, 353 included at least one read mapping to an unexpected species – that is, showed evidence for between-species contamination. Of these, 205 were contaminated by at least two species, and we detected up to eight contaminant species in samples GA08R (Glanville fritillary *Melitaea cinxia*) and GA34L (mosquito *Culex hortensis*). Summing contaminant species across samples, we found that the data set had been affected by at least 782 distinct events of between-species contamination. This is an underestimate, due to the incompleteness of our reference database, our inability to detect contamination between closely related species, and the possibility of multiple events of contaminations of a given sample by a given species. The number of expected *cox1* reads, unexpected *cox1* reads, and contaminant species per sample are available in Additional file 2: Table S2. Reversely, 94 of the 180 species we processed in this project did contaminate at least one sample from another species. Among these, four species contaminated more than 15 distinct samples, and one, king penguin *Aptenodytes patagonicus*, contaminated samples from as many as 11 distinct species (Additional file 4: Figure S1). We found that the mean prevalence of expected *cox1* reads of a species was significantly correlated with the number of individuals it contaminated (*r* = 0.35, *p* < 10^−3^) and with the total number of contaminant reads it contributed (*r* = 0.45, *p* < 10^−4^, log-transformed number of contaminant reads).

### Dubious samples

Two samples resulted in unexpected patterns. Sample GA36K, assigned to species *Mytilus trossulus* (bay mussel), yielded a single *cox1* read that mapped to a *M. trossulus* reference, but >18,000 *cox1* reads that mapped to a sequence from either *M. edulis* or *M. galloprovincialis*, two interbreeding species of European mussels (Fig. 2b, top left dot). By contrast, 99% of *cox1* reads from the other *M. trossulus* sample that we analyzed, GA36L, mapped to a *M. trossulus* reference. The GA36K sample was collected in Seattle, WA, USA, a state in which invasive populations of European mussels are documented [40, 41]. Sample GA36K therefore probably results from an identification error, or reflects *M. galloprovincialis/edulis* mtDNA introgression into *M. trossulus*.

Similarly, sample GA08F, assigned to Glanville fritillary *Melitaea cinxia* (Lepidoptera), did not yield a single *cox1* read that mapped to a *M. cinxia* reference, but >26,000 cox1 reads that mapped to a reference from the Spanish fritillary *Euphydryas desfontainii*. This species is quite divergent from *M. cinxia*, both morphologically and molecularly (*cox1* divergence >25%), so mtDNA introgression and misidentification appear unlikely in this case. According to our records, the GA08F sample came from Aland, Finland, a place where *E. desfontainii* does not occur. We did, however, sample *E. desfontainii*, together with *M. cinxia*, in Morocco. The problem, therefore, probably resulted from sample mislabeling. The GA08F sample very likely belongs to *E. desfontainii* and was mistaken for an *M. cinxia* individual in our published analyses. We checked, however, that our main results are robust to these problems (see final paragraph of the “Results” section).

### Analysis of laboratory metadata

We created a between-species contamination matrix **M** in which cell *m*_*ij*_ contained zero in the absence of evidence for contamination of species *j* by species *i*, one in case of the detected contamination of species *j* by species *i*, and missing data if species *i* and *j* were <5% divergent *cox1*-wise, such that contamination detection was assumed to be unreliable. Here, a single read from any individual of species *i* hitting a reference sequence from species *j* was considered sufficient to attest for an event of contamination of *i* by *j*. Requiring at least ten unexpected reads, instead of just one, yielded qualitatively similar results. The 39 samples from species not represented in our reference *cox1* database were here disregarded, so that sample size was 407 in this analysis. The total number of ones in **M** was 362, and the total number of pairs of species sufficiently divergent such that contamination detection was possible was 27,251, so that the proportion of species pairs for which an event of contamination was detected was *p* = 0.0133.

We focused on five predictors of the probability for two species to be connected by contamination, namely lab_overlap, same_technician, same_shipment, same_flowcell, and same_lane. To calculate the lab_overlap variable, we first defined the processing period of any given species as the period from date of entry into our laboratory to date of last shipment to a sequencing center. For any given pair of species, lab_overlap was defined as the length, in days, of the intersection between the processing periods of the two species. The same_technician variable was a Boolean variable set to one if at least one sample of each of the two considered species was treated by the same person in our laboratory, and to zero otherwise. Similarly, the same_shipment, same_flowcell, and same_lane variables indicated whether at least one sample of each of the two considered species had been shipped on the same day to the same sequencing center, or sequenced on the same flowcell/same lane, respectively.

**Fig. 3:**
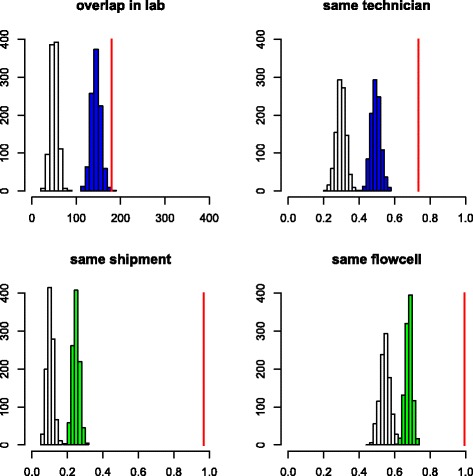
Effect of laboratory metadata on the probability of between-species contamination. Four statistics are shown: lab_overlap (*top left*), same_technician (*top right*), same_shipment (*bottom left*), same_flowcell (*bottom right*). *x*-axis: average value of each statistics. *Vertical red line*: actual data set. *y*-axis: number of randomized data sets (out of 1000). *White histograms*: expected distribution assuming random probability of contamination. *Blue histograms*: expected distribution assuming that contamination is dependent on same_shipment. *Green histograms*: expected distribution assuming that contamination is dependent on lab_overlap and same_technician

We calculated the average value of these variables across all pairs of species for which an event of contamination was attested (Fig. 3, red vertical bars), and compared these to null distributions obtained by shuffling zeros and ones in the contamination matrix (Fig. 3, white histograms, 1000 replicates). More precisely, each cell of a randomized matrix was assigned one with probability *p*, or zero with probability (1 − *p*), with missing data being left unchanged, where *p* = 0.0133 was the overall probability of contamination (see above). We detected a strong and significant effect of each of the five variables: compared to the average species pair, species contaminating each other tended to have a longer period of overlap in our laboratory, to be handled by the same technician, and to be sent the same day and sequenced on the same flowcell. The effect of sequencing center-associated variables was particularly strong. For instance, the probability for two species that were shipped together to be connected by an event of contamination was 0.13, that is, more than ten times the unconditional probability. The same_lane pattern was very similar to same_flowcell and is not shown in Fig. 3.

The five analyzed variables were significantly correlated with each other. We tried to disentangle their effects, and particularly distinguish the influence of our laboratory from that of sequencing centers. To this aim, we compared the observed value of lab_overlap and same_technician to null distributions obtained by reshuffling **M** in a way that controls for the effects of same_shipment (Fig. 3, top, blue histograms). In this analysis, each (*i*, *j*) cell of a randomized matrix was assigned one with probability *p*_*ij*_, or zero with probability (1 − *p*_*ij*_), again leaving missing data unchanged, where *p*_*ij*_ was the probability of contamination knowing same_shipment(*i*, *j*). These were obtained by calculating the proportion of ones in **M** conditional on values 0 or 1 for same_shipment. Similarly, the null distributions of same_shipment and same_flowcell conditional on lab_overlap and same_technician were generated (Fig. 3, bottom, green histograms). The effects of the five variables were still significant in these control analyses: a laboratory effect was detected when controlling for sequencing center-associated variables and a sequencing center effect was detected when controlling for laboratory-associated variables.

To analyze this effect more deeply, we created two synthetic variables summarizing the effect of laboratory (LAB) and sequencing center (CENTER), respectively. The LAB variable was positive when same_technician was true and lab_overlap was >200 days, but negative otherwise. The CENTER variable was negative for pairs of species shipped on distinct dates, but positive otherwise. Regarding species pairs that were sent together, we distinguished pairs sequenced on distinct flowcells (CENTER+), the same flowcell but distinct lanes (CENTER++), and the same lane (CENTER+++). In this analysis we focused on the 97 species for which information on shipment dates, flowcell, and lane numbers was available for all individuals. As far as species sent on distinct dates were concerned (CENTER-), the contamination probability was very low regardless of LAB (Table 1, first line). This seems to be incompatible with the hypothesis of a substantial level of contamination in our laboratory. In contrast, the probability that two species shipped on the same day were connected by an event of contamination was as high as 0.2, and further increased in case of shared flowcell and shared lane (Table 1, lines 2 to 4), reaching values >0.5.

**Table 1:** Effect of laboratory and sequencing center variables on the probability of contamination

	**LAB-** distinct technicians or overlap > 200 days	**LAB +** same technician, overlap < 200 days
**CENTER-** distinct shipments	**0.00089** [3/3368]	**0** [0/360]
**CENTER +** same shipment	**0.20** [89/451]	**0.41** [52/128]
**CENTER++** same flowcell	**0.32** [78/247]	**0.50** [52/104]
**CENTER+++** same lane	**0.43** [69/159]	**0.57** [49/86]

Data are presented as: **contamination probability** [number of contaminated species pairs/total number of species pairs]

Surprisingly, we detected a strong and significant interaction between the LAB and CENTER variables (Table 1). Two species being shipped the same day (CENTER+), overlapping in our laboratory, and being handled by the same technician (LAB+) substantially increased the probability of contamination. We suggest that this is an induced effect resulting from the fact that tubes in shipped boxes were ordered by technician, so that samples processed by the same technician in our laboratory were presumably more likely to be processed together by sequencing centers, and therefore to contaminate each other. To test this hypothesis, we subsampled species in such a way that a single species per technician per shipment was kept, so that no induced effect of same_shipment on same_technician was possible. We found eight events of contamination between the 24 species of the subsample. There was still a significant effect of same_shipment on contamination probability in this subsample, but no effect of lab_overlap or same_technician was detected (Additional file 5: Figure S2), suggesting that the LAB effect conditional on CENTER+ reported in Table 1 was an induced effect. These analyses therefore indicate that the vast majority of the events of between-species contamination we detected occurred in sequencing centers. The results were qualitatively unchanged when a 10% threshold was used, instead of 5%, for the minimal *cox1* divergence between contaminant and contaminated species (Additional file 6: Table S3).

### Laboratory contamination: detailed analysis

Eight events of contamination were detected between species that were not shipped on the same date. Of these, four involved Glanville fritillary *M. cinxia*. This is the one species in our data set that included samples for which data on shipment date are missing (GA08B to GA08F, Additional file 2: Table S2). The three species that contaminated or were contaminated by *M. cinxia* but lacked an attested shared shipment date with *M. cinxia* – Iberian hare *Lepus granatensis*, mountain hare *L. timidus* and ascidian *Ciona intestinalis* A – were shipped the same day, May 26, 2010. It seems therefore possible, not to say probable, that samples GA08B to GA08F were actually sent out for sequencing on May 26, 2010, and that contamination occurred in the sequencing center in this case, too.

Besides these four cases, one detected event of contamination between species not shipped on the same date involved gorgonian *Eunicella cavolini* and European blue mussel *M. galloprovincialis. E. cavolini*, however, shares a shipment date (January 23, 2013) with *M. edulis*, the other species of European mussel, which hybridizes with *M. galloprovincialis* – the two species have very similar haplotypes in our reference *cox1* database. A closer inspection of the data revealed that the single *E. cavolini* sample, GA31L, affected by contamination from *M. galloprovincialis* is the single *E. cavolini* sample that was shipped on January 23, 2013. Eight *cox1* reads from this sample mapped to a *M. edulis* reference and two mapped to a *M. galloprovincialis* reference. In conclusion, only three events of between-species contamination out of 782 can be unambiguously assigned to our laboratory: contamination of European pond turtle *Emys orbicularis* by ascidian *Ciona intestinalis A* and of seahorses *Hippocampus hippocampus* and *H. guttulatus* by each other.

### Within-species contamination

The above analyses suggest that there was substantial contamination in this project, and primarily involves samples that were shipped together. This is worrisome because samples from distinct individuals of the same species, between which contamination is most problematic and difficult to detect, were typically sent together. To quantify the amount of within-species contamination, we examined the prevalence as the minor state (“errors”) at homozygous genotypes of alleles segregating in the sample. First focusing on homo-quartets (i.e., positions at which the read count for the major state was >40 and the read count for the minor state equaled 1) that occurred at monoallelic positions, we determined **P**, the error matrix in the absence of contamination. This was done separately for each of the 39 species of the sample in which at least four individuals were sequenced. Note that in this study we did not use strand information, so we could not distinguish between X → Y and X* → Y* errors, where X* is the complementary of base X.

Error matrices revealed two main features. First, the A → C or T → G errors were often more frequent than the other three transversion-type errors, namely A → T or T → A, C → G or G → C, and C → A or G → T. The ratio of A → C or T → G to other transversion-type errors varied between 0.29 and 0.79 among species (correcting for base composition), when a ratio of 0.67 would be expected under random error. This is consistent with documented error biases of the Illumina technology [42, 43]. Second, transition-type errors, C → T or G → A and T → C or A → G, were typically more numerous than expected. The ratio of transition-type to transversion-type errors varied from 0.47 to 1.14 among species (correcting for base composition, median = 0.79), when the expected ratio would be 0.5 under random error, and <0.5 according to [43]. Knowing that DNA polymerases typically generate more transition-type than transversion-type errors, this result suggests that a fraction of the sequencing errors affecting our data was introduced prior to sequencing, presumably at the PCR step during library construction.

We then considered homo-quartets occurring at biallelic positions, where two alleles segregate at substantial frequency. Here, we only considered the 33 species in which at least 50 such homo-quartets were found. We asked whether the minor state at such homo-quartets tended to correspond with the other segregating allele more often than expected based on **P**. We found that the relative prevalence of the other segregating allele was above its expected value in all 33 species. The index of allele leakage, λ, varied from 0.19 to 8.5*,* when λ = 0 would be expected in the absence of contamination. This analysis therefore indicates that within-species contamination is widespread in our dataset and probably affects all the sequenced species.

We investigated the influence of laboratory metadata, and particularly the date of shipment to sequencing centers, on the prevalence of within-species contamination. To this end, we focused on the 12 species of our data set in which not all samples were shipped the same day – that is, most often at two distinct dates, and up to four dates in the blue tit *Parus caeruleus*. In these species, we measured λ', the index of allele leakage between samples sent on different dates. This was achieved by only considering homo-quartets occurring at positions that were biallelic across the whole sample of individuals, but monoallelic in the subsample of individuals shipped the same day as the focal individual (Additional file 7: Figure S3). This analysis could not be performed species by species due to the small number of relevant homo-quartets per species. We therefore pooled homo-quartets across the 12 species, still accounting for species-specific error matrices **P**, and obtained an index of allele leakage between samples sent on different dates of λ' = 0.59. This figure was twice as small as the index calculated as above, that is, irrespective of shipment date, which for these 12 pooled species was λ = 1.21, demonstrating an effect of same_shipment on the prevalence of within-species contamination.

### Contamination-aware SNP calling

To assess the robustness of our published results to the problem of within-species contamination, we re-called SNPs and genotypes using a modified method accounting for allele leakage between individuals. Compared to our original SNP-calling method, a parameter γ was added, which represents the probability that a read originates from another individual of the sample. Three arbitrary values of γ were used: 0.05, 0.1, and 0.2. Contamination-aware SNP calling was applied to the 39 species of our sample in which at least four individuals were available. Classical population genomic statistics were calculated from this data set using the same pipeline as in [18]. To save computational time, SNP calling was applied to reduced data sets consisting of exactly one million positions per species, instead of the 1.8–27 million positions in full data sets.

**Fig. 4:**
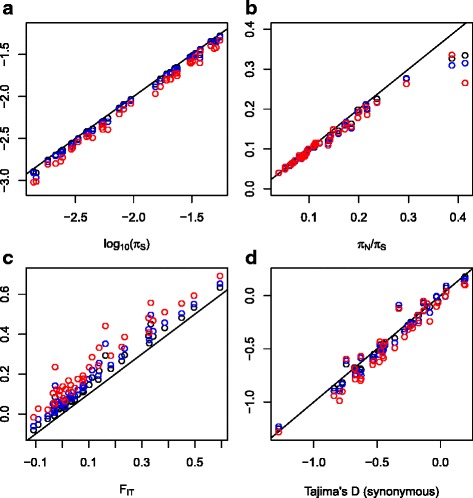
Robustness of population genomic estimates to contamination-aware single-nucleotide polymorphism (SNP) calling. **a** Synonymous diversity π_S_; **b** ratio of non-synonymous to synonymous diversity, π_N_/π_S_; **c** F_IT_; **d** Tajima’s D, synonymous SNPs only. Each *dot* represents a species. *x*-axis: estimates obtained assuming no contamination. *y*-axis: estimates obtained from contamination-aware SNP calling. *Black dots*: γ = 0.05; *blue dots*: γ = 0.1; *red dots*: γ = 0.2 synonymous diversity πS; *top right*: π_N_/π_S_ ratio; bottom left: F_IT_; bottom right: Tajima’s D, synonymous SNP’s only

We found that the number of called SNPs and the estimate of π_S_, the genetic diversity at synonymous positions, decreased with increasing γ (Fig. 4a). This was expected: contamination spuriously increases heterozygosity by moving alleles around. The relative bias was substantial – the median ratio of corrected to uncorrected π_S_ was 0.90 when γ was 0.1, and 0.81 when γ was 0.2. The relative bias, however, was fairly constant across species, and much smaller that the between-species differences in π_S_, suggesting that our published comparative analyses of π_S_ across species [17, 19, 21, 22] are robust to within-species contamination. We checked that the correlation reported by Romiguier et al. [21] between π_S_ and species life history traits were still valid after control for contamination. We found that the correlation coefficient between log-transformed π_S_ and log-transformed longevity was very similar in all four analyses, that is, between −0.517 and −0.524, the most negative coefficient being obtained when γ = 0.1. Similarly, the relationship between log-transformed π_S_ and log-transformed propagule size [21] was very robust to changes in γ (correlation coefficient between and −0.772 and −0.758, minimal value when γ = 0).

The ratio of non-synonymous to synonymous diversity, π_N_/π_S_, was only slightly modified when we controlled for contamination (Fig. 4b), the median relative bias being close to 0.96 for all three positive values of γ. The synonymous (Fig. 4d) and non-synonymous Tajima’s D, a statistic measuring the departure of the distribution of minor allele frequency from the standard coalescent, were also only moderately affected. These two results suggest that published inferences based on π_N_/π_S_ and site-frequency spectra [18, 27] are presumably robust enough to within-species contamination.

The F_IT_ statistics measures the excess of individual homozygosity compared to Hardy-Weinberg expectations. A positive F_IT_ is expected in cases of inbreeding and/or population substructure. Figure 4c shows that our F_IT_ estimate is particularly sensitive to contamination issues. Controlling for contamination resulted in a substantial increase in F_IT_ in all the analyzed species, reflecting the fact that within-species contamination tends to increase individual heterozygosity. In our uncorrected analysis (γ = 0), a negative estimate of the genome-average F_IT_ was obtained in nine species [21]. This is an unexpected result, given that processes leading to heterozygote excess, such as balancing selection, are presumably limited to a small fraction of the genome [44]. In our contamination-aware analyses, a negative F_IT_ was obtained in just four, two, and one species when γ was set to 0.05, 0.1, and 0.2, respectively, suggesting that within-species contamination might explain, at least partly, our previously unexpected report of negative estimates of F_IT_ [21]. Harvest ant *Messor barbarus* was not included in this analysis because the genome-average F_IT_ is very negative in this species as a consequence of its peculiar mating system, such that worker individuals are highly heterozygous [45].

We have not commented on F_IT_ estimates in our published analyses, with the exception of [19], in which the lack of detectable population substructure (i.e., low F_IT_) in the giant Galapagos tortoise *Chelonoidis nigra* provided evidence against the definition of as many as 12 species in this taxon [46]. This result was here corroborated: *C. nigra* is one of the two species still showing a slightly negative F_IT_ estimate after correction for contamination. We have, however, published a couple of analyses assessing the prevalence of hybridization and gene flow between diverged species or populations [20, 28, 30]. These results should be confirmed by reproducing the analyses using contamination-corrected data.

We compared for each species the likelihoods of the four considered values of γ. The maximally likely γ, which we called γ*, was 0 in ten species, 0.05 in 15 species, 0.1 in five species, and 0.2 in nine species. We detected a strong effect of species diversity on γ* : the median π_S_ was 0.034 among species for which γ* was 0, but 0.003 among species for which γ* was 0.2. This was unexpected and probably reflects the existence of factors that confound contamination detection (see section 3 of the Discussion "Modeling contamination").

Finally, we reproduced the analyses of Romiguier et al. (2014) [21], accounting for the dubious GA36K and GA08F samples. The published relationships between genetic diversity and species life history traits were robust to the exclusion of *M. trossulus* and *M. cinxia*: the correlation coefficient between π_S_ and propagule size was almost unchanged compared to the uncorrected analysis (0.766 vs. 0.771), whereas the correlation coefficient between π_S_ and longevity was slightly increased (0.594 vs. 0.569), as was the case for correlations between the π_N_/π_S_ ratio and life history traits. We recalculated population genomics statistics in *M. cinxia* after excluding individual GA08F, that is, based on just nine individuals instead of ten. Excluding GA08F resulted in a substantial decrease in genome-average π_S_ (0.025 vs. 0.034), π_N_ (0.0027 vs. 0.0032), and F_IT_ (0.38 vs. 0.52). Correlation coefficients with life history traits, however, were hardly affected by this correction.

## Discussion

Here, we analyzed the prevalence and impact of between-species and within-species contamination in an RNAseq project involving 446 samples from 116 distinct species of animals. We focused on cross-contamination and contamination from model animals. We did not investigate contamination from, for example, microbes, which can be highly problematic, too [15]. This is in part because our experimental process targets polyA-containing RNAs, which filters out the bulk of bacterial mRNAs, and in part because a BLAST search that was performed in a previous study [21] indicated very low levels of microbial contamination in our final sets of contigs. Our analysis indicates that cross-contamination was widespread: approximately 80% of our samples showed evidence of contamination by a foreign species, and traces of within-species contamination were detected in all the species we analyzed. Contamination in this project was not an accident, it was a pattern.

A single unexpected *cox1* read was taken as sufficient to document an event of between-species contamination in our analysis. This might appear too liberal a criterion: contamination at such low levels is very unlikely to affect the analyses or the conclusions. The near-zero detection of contaminant reads from companion species, however, demonstrates that the unexpected *cox1* reads we uncovered do not result from environmental contamination or experimental noise, but indeed trace transfers of genetic information from sample to sample. Even at very low prevalence, therefore, unexpected reads do provide relevant information on when and how events of contamination have happened. Our results were qualitatively unchanged when we only counted events of contamination supported by at least ten reads (Additional file 8: Figure S4).

### Contamination occurred in sequencing centers

We uncovered indirect evidence that the vast majority of the events of cross-contamination occurred in sequencing centers. This was attested to by the very strong effect of same_shipment on the probability of between-species contamination, and confirmed by the reduced within-species allele leakage when only samples sent on different dates were considered. Roughly 15% of species pairs sharing a common shipment were connected by at least one event of contamination, and we detected only three events of contamination between species that were not shipped together. In this project, libraries were constructed in the sequencing centers. This step involves PCR amplification and might be more prone to contamination than RNA extraction, purification, and quantification, which were achieved in-house. It might also be that sequencing centers were simply less careful than our technicians about contamination. Sequencing center SC2 only handled two shipments, and SC3 only one, so we do not have sufficient power to compare centers in this analysis.

In principle, contamination could occur during the library preparation stage through physical transfer of material, or during the sequencing stage through mis-tagging – That is, when the identifier assigning a read to its source sample is in error. In this project, we used simple indexing of samples, which can result in a non-negligible rate of sample misidentification [47]. We detected a strong effect of shipment, and on top of this a significant effect of flowcell and lane identity (Table 1), suggesting that contamination occurred during both stages. However, in the absence of data about which samples were handled together during library preparation, it is difficult to firmly conclude at which experimental steps contamination most often occurred – especially if libraries prepared together were more likely to be sequenced in the same flowcell/lane, as might well have been the case.

It should be noted that our index of allele leakage λ was still significantly higher than zero in the control analysis when only samples sent at different dates were considered. This might indicate that a fraction of the events of within-species contamination did occur in our laboratory. Alternatively, λ might be inflated by processes different from contamination, such as hotspots of systematic errors [43], mosaicism [48], hidden paralogy, and variable expression level between alleles and individuals [17, 18]. Approaching and quantifying within-species contamination is actually a difficult problem, especially with RNAseq data, because a number of distinct processes can potentially generate asymmetric read counts. Families of recently duplicated genes are particularly tricky in this respect: they will yield an unpredictable number of contigs after de novo assembly, which will each attract a fraction of the reads of the distinct individuals at the mapping step. This might generate patterns similar to the ones shown in Fig. 1, confounding within-species contamination detection (see below).

### Modeling contamination

We introduced a modified SNP-calling method that accounts for within-species contamination by assuming that a fixed fraction of the observed reads originates from other individuals of the sample. Estimates of the classical population genomic statistics were affected to various extents by this correction, depending on the assumed contamination rate γ. F_IT_ was particularly sensitive to γ, calling for caution as far as studies of population substructure and gene flow are concerned. The effect of γ on population genomic estimates was essentially homogeneous among species, suggesting that our published comparative analyses are reasonably robust, as we explicitly checked in some cases.

In these control analyses, γ was fixed to arbitrary values. When we tried to estimate the contamination rate in the maximum-likelihood framework, together with the sequencing error rate and transition/transversion ratio, we obtained estimates of γ that were negatively correlated to species genetic diversity. We suspect that this might reflect the confounding effect of hidden paralogy, when the reads corresponding to two paralogous genes map to a single reference cDNA due to erroneous assembly. Hidden paralogy tends to mimic contamination by resulting in sites at which all individuals carry similar read counts for two distinct “alleles” [18]. Such sites tend to inflate the estimate of γ, particularly in low-diversity species, where the ratio of spurious to correct SNPs is maximal. Read counts in NGS projects are typically over-dispersed compared to the multinomial distribution that is assumed by SNP-calling methods, and contamination is one out of several sources of over-dispersion (see above). To distinguish between these various effects is a methodological challenge that would require further developments. One consequence is that we do not know which values of γ in our analyses are closer to the true contamination rates – which perhaps differ between species and between pairs of individuals. It is therefore premature to draw conclusions on the quantitative impact of within-species contamination on population genomic statistics based on this analysis. We still believe that the newly introduced γ parameter, which likely captures a combination of undesired effects, tends to improve the accuracy of predicted SNPs and genotypes – as reflected by the positive values of genome-average F_IT_ we obtained when assuming non-zero γ. For this reason, this approach and recently published related approaches [10, 49] deserve to be further developed.

## Conclusions

Are our results generalizable to other NGS-based population genomic/molecular ecology studies? We are not sure, mainly because sequencing centers were critical in the contamination patterns we detected, and our sampling of sequencing centers was poor. There is, however, no reason to a priori believe that the patterns we uncovered here will not apply to other studies – particularly those having relied on SC1 in 2009–2014. The three shipments addressed to centers SC2 and SC3 were not devoid of contamination, so the problem is probably not specific to SC1. Can guidelines for avoiding contamination be deduced from this analysis? Possibly not, again because sequencing centers were critical and we have no control over, or even knowledge of, their detailed experimental processes. We do, however, still make a number of recommendations. First, we suggest taking the cost of potential contamination into account when deciding to subcontract, or not, part of a research project in molecular biodiversity. Second, if samples have to be shipped for sequencing, we would suggest, whenever possible, sending together samples that are as genetically divergent as possible, such that contamination would be both easier to detect and less problematic. Third, when possible, we would suggest sending replicated samples, preferably on distinct dates, as controls for contamination. This can be expensive but is a direct way to identify and clean contaminant sequences, and measure their prevalence. Finally, we suggest that bioinformatic pipelines for NGS-based population genomic data should be further developed/improved in order to account for the probable existence of between-species and within-species contamination.

## Additional files

Additional file 1: **Table S1.** List of species and laboratory metadata. (XLS 55 kb)

Additional file 2: **Table S2.** List of samples, shipment dates, and prevalence of expected and unexpected *cox1* reads. (XLS 101 kb)

Additional file 3: Alignment of reference *cox1* sequences used in this study. The fragment we used corresponds to positions 6189–6539, Cambridge reference sequence. (TXT 241 kb)

Additional file 4: Figure S1. Contaminant species-associated statistics. Each dot is for a contaminant species, that is, a species for which at least one *cox1* read was found in a sample from another species. *x*-axis: number of contaminated species (median = 2). *y*-axis, bottom: number of contaminated individuals (median = 4). *y*-axis, top: number of contaminant reads (median = 65). (PDF 40 kb)

Additional file 5: Figure S2. Effect of laboratory metadata, one species per technician per date. See legend to Fig. 3. Here, a single species per technician per shipment was kept, removing any possible induction by same_shipment of a same_technician effect on the probability of between-species contamination. No significant effect of laboratory-associated variables is detected in this control. (PDF 5 kb)

Additional file 6: Table S3. Effect of laboratory and sequencing center variables on the probability of contamination, with the minimal *cox1* divergence between contaminant and contaminated species being set to 10% instead of 5%. (XLS 6 kb)

Additional file 7: Figure S3. Homo-quartet analysis of contamination between shipments. See legend to Fig. 1. Here we assume that Ind_1_ and Ind_2_ have been shipped together on a date different from the shipment date of Ind_3_ and Ind_4_. We want to specifically assess the prevalence of contamination between individuals shipped at different dates. Here, Pos_4_ is not considered because the {Ind_1_, Ind_2_} group is not monoallelic, so that contamination could involve two individuals shipped together. Only Pos_5_ is identified as a candidate for between-shipment contamination. (PDF 27 kb)

Additional file 8: Figure S4. Effect of laboratory metadata, at least ten reads per contaminant. See legend to Fig. 3. Here, at least ten unexpected *cox1* reads were required to call a contaminant, instead of one read in the main analysis. (PDF 5 kb)
