# Supplementary figures and images for: Patterns of cross-contamination in a multispecies population genomic project: detection, quantification, impact, and solutions
Source: BMC Biol. 2017 Mar 29;15:25. doi: 10.1186/s12915-017-0366-6 (PMC5370491; doi:10.1186/s12915-017-0366-6)

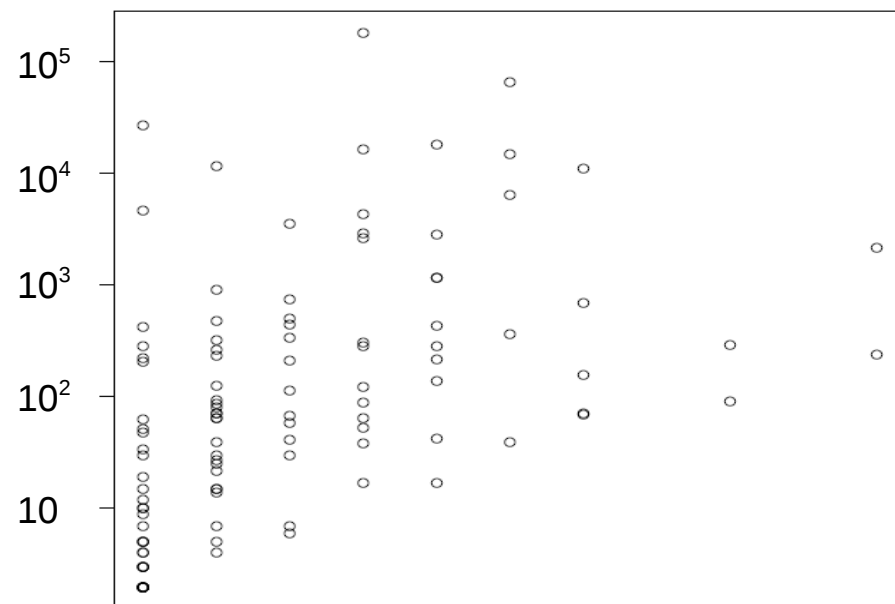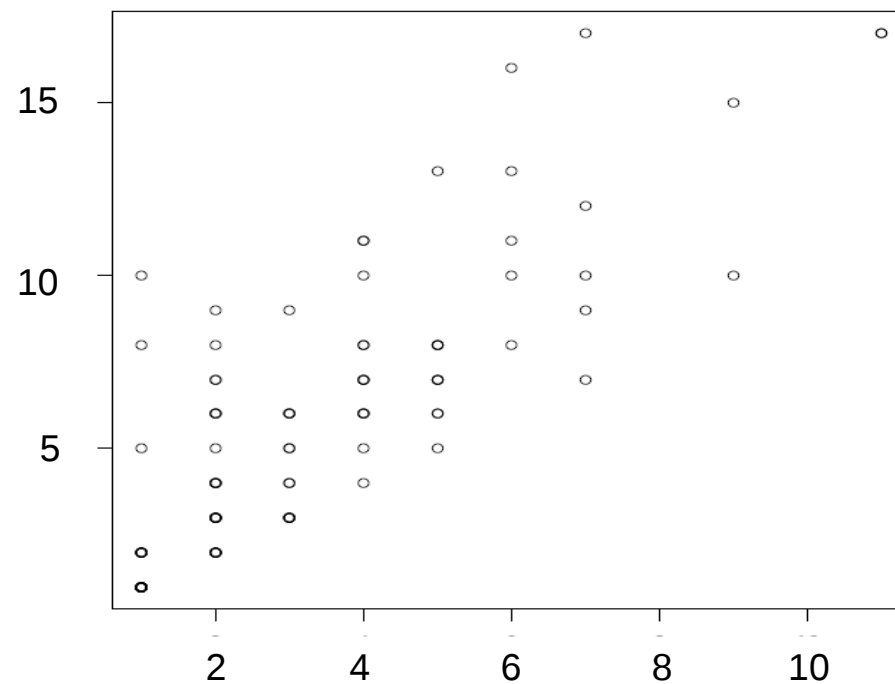

## # contaminated species

Supplement: Supplementary file 4 — Contaminant species-associated statistics. Each dot is for a contaminant species, that is, a species for which at least one cox1 read was found in a sample from another species. x-axis: number of contaminated species (median = 2). y-axis, bottom: number of contaminated individuals (median = 4). y-axis, top: number of contaminant reads (median = 65). (PDF 40 kb) [file 12915_2017_366_MOESM4_ESM.pdf]

**overlap in lab**

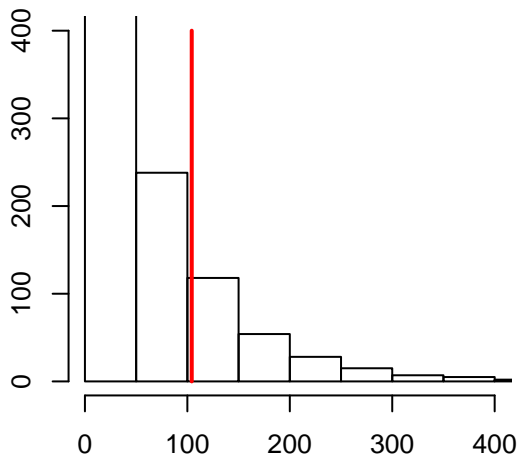

**same technician**

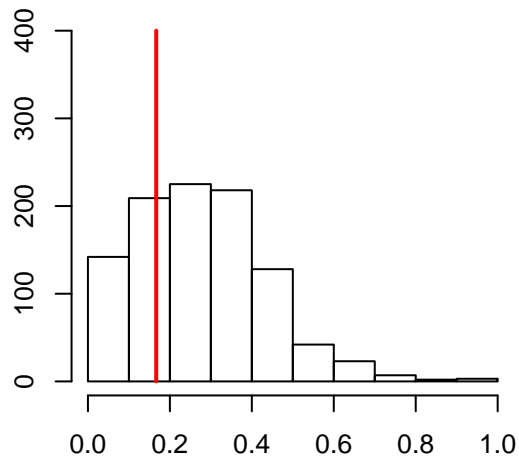

**same shipment**

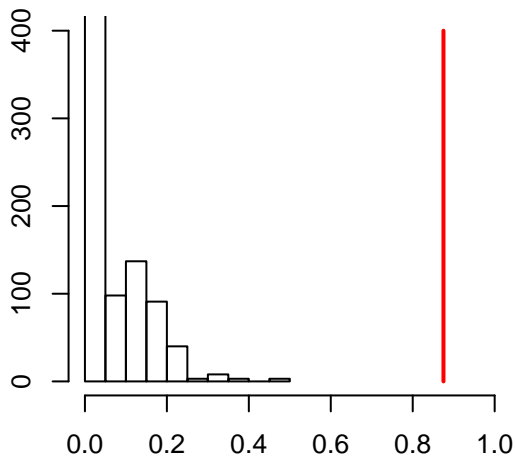

**same flowcell**

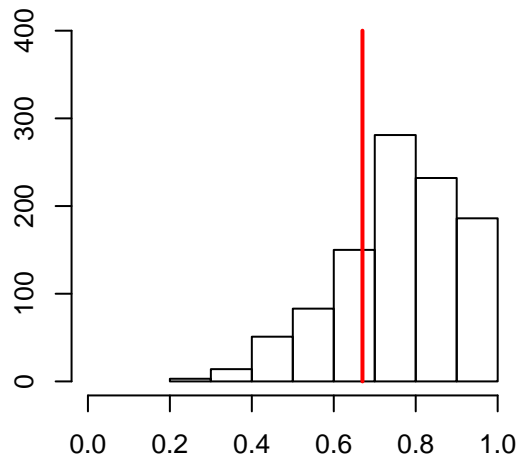

Supplement: Supplementary file 5 — Effect of laboratory metadata, one species per technician per date. See legend to Fig. 3. Here, a single species per technician per shipment was kept, removing any possible induction by same_shipment of a same_technician effect on the probability of between-species contamination. No significant effect of laboratory-associated variables is detected in this control. (PDF 5 kb) [file 12915_2017_366_MOESM5_ESM.pdf]

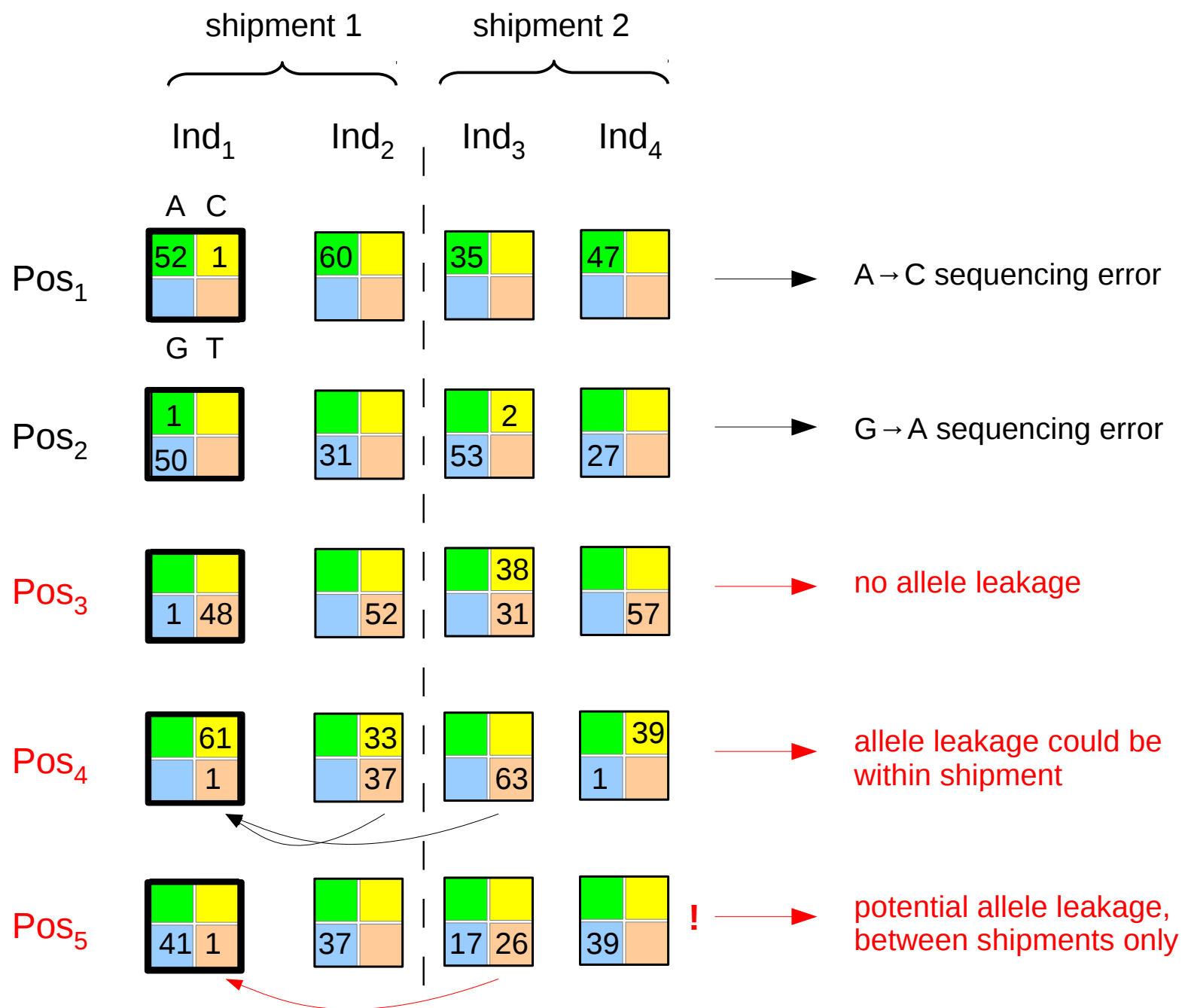

Supplement: Supplementary file 7 — Homo-quartet analysis of contamination between shipments. See legend to Fig. 1. Here we assume that Ind1 and Ind2 have been shipped together on a date different from the shipment date of Ind3 and Ind4. We want to specifically assess the prevalence of contamination between individuals shipped at different dates. Here, Pos4 is not considered because the {Ind1, Ind2} group is not monoallelic, so that contamination could involve two individuals shipped together. Only Pos5 is identified as a candidate for between-shipment contamination. (PDF 27 kb) [file 12915_2017_366_MOESM7_ESM.pdf]

**overlap in lab**

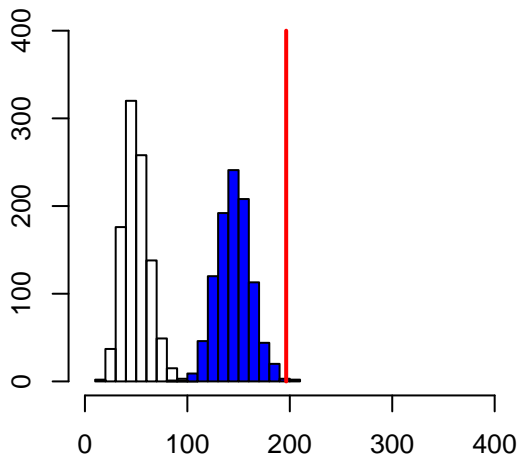

**same technician**

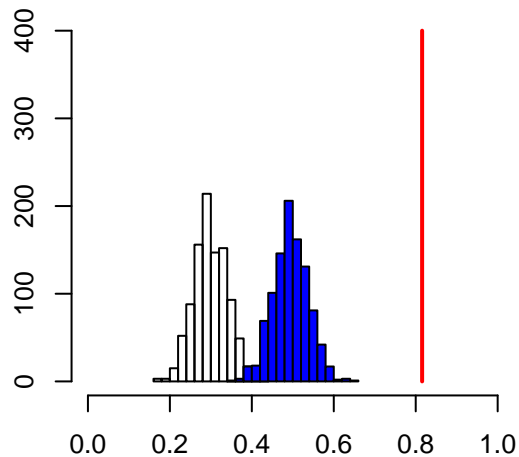

**same shipment**

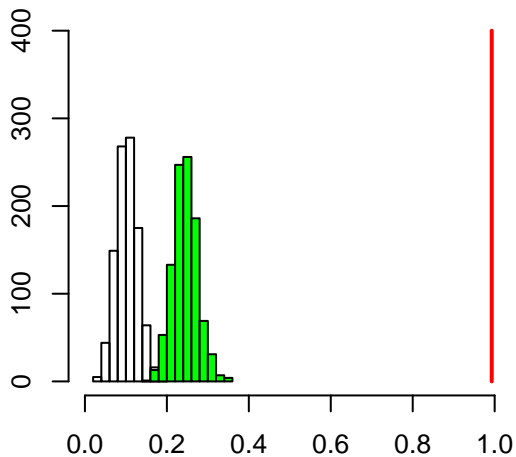

**same flowcell**

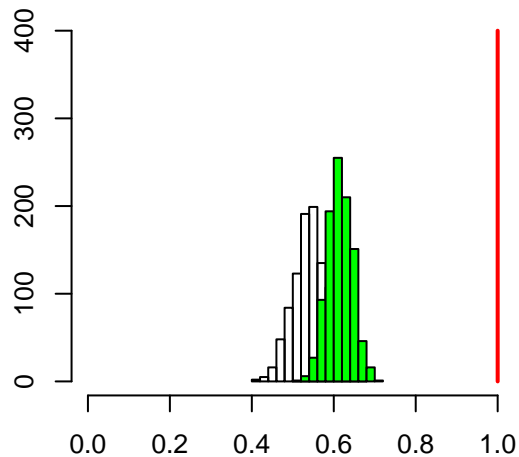

Supplement: Supplementary file 8 — Effect of laboratory metadata, at least ten reads per contaminant. See legend to Fig. 3. Here, at least ten unexpected cox1 reads were required to call a contaminant, instead of one read in the main analysis. (PDF 5 kb) [file 12915_2017_366_MOESM8_ESM.pdf]
